# Editorial: Cardiovascular involvement in autoimmune diseases, volume II

**DOI:** 10.3389/fcvm.2024.1352268

**Published:** 2024-01-31

**Authors:** Sophie I. Mavrogeni, Lambros Fotis, Marco Matucci-Cerinic

**Affiliations:** ^1^ Onassis Cardiac Surgery Center, Kapodistrian University of Athens, Athens, Greece; ^2^ Third Department of Pediatrics, Attikon Hospital, Athens, Greece; ^3^ Unit of Immunology, Rheumatology, Allergy and Rare Diseases (UnIRAR), St Rafael University Hospital, Milan, Italy

**Keywords:** autoimmune rheumatic disease (ARD), myocarditis, cardiomyopathy, autoimmune, immunomodulation

**Editorial on the Research Topic** Cardiovascular involvement in autoimmune diseases, volume II

Current therapeutic approaches for autoimmune rheumatic diseases (ARDs) have led to a reduction of disease-associated mortality in these patients. Nevertheless, the life expectancy of patients with ARDs still remains lower, compared with that of the general population (1). This is mainly due to the high incidence of cardiovascular disease (CVD) (2–7).

CVD in patients with ARDs is the result of various pathophysiologic mechanisms including systemic, myocardial, vascular inflammation, accelerated atherosclerosis, myocardial ischemia, due to micro- of macro-vascular lesions, replacement and/or diffuse fibrosis (8, 9). Irrespective of the underlying clinical syndrome, cardiovascular symptoms in patients with ARDs are usually under-estimated or not promptly recognized. Usually, they are attributed to the underlying systemic disease and not to involvement of the cardiovascular system. Notably, clinically overt CVD indicates advanced cardiac involvement and carries a poor prognosis (10). Furthermore, the development of CVD may already have started two or even three years before the diagnosis of ARD (10). For these reasons, scientific understanding regarding the pathophysiology of CVD in patients with ARDs, as well as tailored treatment approaches for each individual subtype of ARD are currently needed.

In this dedicated issue of “Frontiers in Cardiovascular Medicine”, thoughts about autoimmune myocarditis and atrioventricular block in foetuses of mothers with systemic lupus erythematosus are discussed.

In the paper by Wu et al., the authors report that inhibition of necroptosis, apoptosis, or autophagy in a model of experimental autoimmune myocarditis reduced myocardial inflammatory cell infiltration and improve cardiac function. Since apoptosis or autophagy is involved in many important cellular aspects, instead of suppressing these two major cell death processes, Nec1 can be developed as a potential therapeutic target for inflammatory myocarditis (Wu et al.). This study provides some novel insights regarding a potential future treatment approach for autoimmune myocarditis. However, it remains to be investigated whether such treatments can still provide clinical benefits when added to the current standard of care.

In a case report presented by Tang et al. (11) a prenatal diagnosis and treatment of fetal autoimmune-associated first-degree atrioventricular block was discussed. According to the authors, prenatal treatment for fetal autoimmune-associated first-degree atrio-ventricular block could be an alternative, in parallel with strict surveillance and timely treatment of both mother and fetus. Nevertheless, the authors only point towards a possible prevention of this complication in the fetus and more work is required to establish causality in this context.

Lastly the paper by Zhao et al. (12) presented some additional findings from a small case series of four pregnant women, whose foetuses developed first-degree antrioventricular block. This scientific work further adds to the aforementioned paper of Tang et al. (11) Currently used treatments include corticosteroids, hydroxychloroquine, intravenous immunoglobulin (IVIG), beta-sympathomimetic agents, and even plasma exchange. However, approaches for preventing the progression/recurrence of a fetal atrio-ventricular block are still controversial. In this paper, the authors describe their experience regarding five foetuses (including one pair of twins) diagnosed with first degree atrioventricular block secondary to maternal anti-SSA/Ro autoantibody seropositivity. The foetuses were successfully treated with a combination of dexamethasone and hydroxychloroquine. This study provided some better-quality evidence in a higher number of patients, but larger multi-center randomized control trials are required to firmly establish the appropriate diagnostic approach in these cases.

Although the hot point of CVD in ARDs is not completely covered in this issue, we believe that some interesting aspects discussing challenging diagnostic and therapeutic aspects of these diseases were presented.
